# The Relieving Effects of a Polyherb-Based Dietary Supplement ColonVita on Gastrointestinal Quality of Life Index (GIQLI) in Older Adults with Chronic Gastrointestinal Symptoms Are Influenced by Age and Cardiovascular Disease: A 12-Week Randomized Placebo-Controlled Trial

**DOI:** 10.1155/2021/6653550

**Published:** 2021-09-10

**Authors:** Gang Xu, Guoqiang Xing, Bing Zhang, Jingfen Zhu, Yong Cai, Tian Shen, Jianyu Rao, Rong Shi, Zhaochun Cao, Tuong Nguyen

**Affiliations:** ^1^Department of Community Health and Behavioral Medicine, School of Public Health, Shanghai Jiao Tong University School of Medicine, Shanghai 200025, China; ^2^The Affiliated Hospital and the Second Clinical Medical College of North Sichuan Medical University, Nanchong Central Hospital, Nanchong 637000, China; ^3^Lotus Biotech.com LLC, John Hopkins University-MCC, 9601 Medical Center Drive, Rockville 20850, Maryland, USA; ^4^Department of Pathology and Laboratory Medicine, David Geffen School of Medicine, University of California at Los Angeles, Los Angeles 90095, California, USA; ^5^School of Public Health, Shanghai University of Traditional Chinese Medicine, Shanghai 201203, China; ^6^Jiaxing Subdistrict Community Health Service Center, Hongkou District, Shanghai 200086, China; ^7^Department of Research, DRM Resources, 1683 Sunflower Avenue, Costa Mesa, CA 92626, USA

## Abstract

Chronic gastrointestinal symptoms (CGS) negatively affect the quality of life in about 15–30% of the population without effective drugs. Recent studies suggest that dietary supplement may improve CGS, but inconsistent results exist. The goal of this study is to evaluate the effect of a polyherbal-based supplement ColonVita on the gastrointestinal quality of life index (GIQLI) in 100 old adults with CGS (63.1 ± 9.6 years) who were randomly assigned to daily ColonVita or placebo tablets (*n* = 50/group) for 12 weeks in a double-blind, randomized controlled trial design. No significant fibrdifferences were found between ColonVita and placebo in the baseline total GIQLI score (101.12 ± 16.87 vs. 101.80 ± 16.48) (*P* > 0.05) or postintervention total GIQLI score (114.78 ± 9.62 vs. 111.74 ± 13.01) (*P* > 0.05). However, ColonVita significantly improved 16 scores of the 19 core GI symptoms compared with 10 items improved by placebo. The ColonVita group significantly improved the remission rate of 5 core GI symptoms compared to placebo and significantly improved the total GIQLI scores (118.09 ± 7.88 vs. 109.50 ± 16.71) (*P* < 0.05) and core GI symptom scores (64.61 ± 3.99 vs. 60.00 ± 8.65) (*P* < 0.05) in people ≥60 years of age (*n* = 49) but not in those under 60 y (*n* = 51). ColonVita significantly improved the total GIQLI scores and core GI symptom scores in people without cardiovascular diseases (CVD) (*n* = 56) (116.74 ± 9.38 vs. 110.10 ± 14.28) (*P* < 0.05) and (63.11 ± 4.53 vs. 59.93 ± 8.03) (*P*=0.07), respectively, but not in those with CVD (*n* = 44). Thus, ColonVita was beneficial for old adults with CGS, especially those ≥60 years of age and without CVD. Because a heterogenous pathogenesis of CGS-like irritable bowel syndrome (IBS) and inflammatory bowel disease (ISD) is differentially associated with CVD, different comorbidities may have influenced the outcomes of different trials that should be controlled in further studies.

## 1. Introduction

Chronic gastrointestinal symptoms (CGS) include a range of medical conditions from irritable bowel syndrome (IBS) to inflammatory bowel disease (IBD), ulcerative colitis (UC), and Crohn's disease (CD) that affect about 15–30% of the general population. The common symptoms of CGS include inflammation, postprandial abdominal pain, dyspepsia, abdominal distention (bloating), anorexia, heartburn, vomiting, constipation, and chronic diarrhea. The impact on the overall quality of life is the most significant complication of CGS [1].

CGS used to be more common in industrialized countries that have steadily increased since the middle of 20th century but remains low in developing countries [2]. However, by the turn of 21st century, CGS have become a global disease as more people in newly industrialized countries adapted to the life styles of the industrialized countries. A recent National Institutes of Health- (NIH-) sponsored National GI Survey shows that an estimated 61% of the US population had ≥1 GI symptoms in the past week, with the heartburn/reflux (30.9%), abdominal pain (24.8%), bloating (20.6%), diarrhea (20.2%), and constipation (19.7%) as the most common symptoms and nausea/vomiting (9.5%), dysphagia (5.8%), and bowel incontinence (4.8%) as the less common symptoms [3]. According to US Center for Disease Control (CDC), there are more than 3 million US adults who had IBD in 2015, and the number is increasing each year. The cases of IBD in China has increased from 1047991 in 1990, to 2665081 in 2017, with a 2.9% annual percentage change (APC) [2, 4].

The pathogenesis mechanism of increased global CGS prevalence is complex and heterogenous. CGS may occur in young and old people due to changes in the intestinal microenvironment or weakened physiological function of the digestive and absorption systems [5]. The contract and relaxation of the muscles lining the intestines to move food along the digestive tract can also be weakened by venous thromboembolism (VTE) and ischemic colitis that result in intermittent abdominal pain/discomfort, altered bowel patterns, and abdominal bloating/distension [6].

Genetic, gender, and dietary may contribute to CGS. Psychological distress, anxiety, depression, and childhood adversity are risk factors of CGS. CGS may also occur due to changes in the intestinal microenvironment and inflammatory status induced by stress and dietary pattern changes associated with industrialization from plant-based fiber-rich diet to proinflammatory high animal fat diet, herbicide/pesticides/antibiotics residuals in food, and genetically modified food or food preservatives that may inhibit or disrupt intestinal microbes, thus compromising the physiological function of the digestive and absorption systems [4, 5, 7–9].

Dietary factors such as milk/milk products, wheat products, caffeine, and fried and smoked food may trigger CGS and VTE [10–20], which are strongly related to obesity, CGS, and cardiovascular diseases [21, 22] that can be controlled by adapting the Dietary Approaches to Stop Hypertension (DASH) diet that is rich in fruits, vegetables, and dairy protein but low in saturated and total fat [23–26].

Current opinion is that developing effective dietary intervention is more important than studying the etiology of CGS [27]. Although the FDA has approved multiple agents for treatment of CGS (laxatives as the anticonstipation agent; loperamide, diphenoxylate, and atropine as antidiarrheal agents; lubiprostone as a chloride channel activator for women with IBS constipation; alosetron hydrochloride as a serotonin 5-HT3 antagonist for women with severe diarrhea-predominant IBS; belladonna alkaloids/phenobarbital, hyoscyamine, dicyclomine, propantheline, and peppermint oil as antispasmodics for abdominal cramps and associated diarrhea; rifaximin as antibiotics for concurrent small bowel bacterial overgrowth; fluoxetine, citalopram, sertraline, desipramine, amitriptyline, venlafaxine, and duloxetine as antidepressants to relieve gut pain and psychological distress, anxiety, and depression) and for IBD (aminosalicylates, corticosteroids (prednisone), immunomodulators, and biologics), these agents do not cure CGS.

Recent studies suggest that dietary or herbal supplements may balance beneficial bacteria in the intestinal track, ease bowel movement, and prevent constipation of IBS [10–12, 28, 29]. Coadministration of multiple medicinal herbs is a common practice in traditional Chinese medicine for multitargeting and for improving bioavailability and efficacy of the therapeutic ingredients. ColonVita is a dietary supplement consisted of the blend of *Aloe* extract, acai tropical fruit extract, citrus bioflavonoids, peppermint leaves extract, magnesium hydroxide, and vitamin C. ColonVita has been used in America and other countries for more than ten years at the time of this study due to its potential properties of nourishing, relieving, lubrication of the gastrointestinal system, stimulating intestinal neural peristalsis, softening the feces, stimulating hepatic secretion of detoxifying enzymes, and controlling *Helicobacter pylori* (*H. pylori*) function from the activities of its key ingredients briefly described.

*Aloe vera* is a plant with antioxidant, anti-inflammatory, antitoxicity, analgesic, and antidiabetic properties and is used in traditional Chinese medicine for treatment of constipation and colitis. *Aloe vera* mitigates dextran sulfate sodium-induced rat ulcerative colitis by potentiating the colon mucus barrier [30]. A pilot randomized trial showed that *Aloe vera* was more effective (42%) than placebo (28%) in relieving IBS symptoms of 58 IBS patients at the trend level [18], and 8-week intervention with 30 ml *Aloe vera* administered twice daily significantly decreased pain, discomfort, and flatulence in 33 individuals with constipation-predominated refractory IBS [31].

Acai is a phytochemical plant with antioxidant, anti-inflammatory, and antigenotoxic effects [32–36]. Dried extract of acai berries improves ethanol-induced ulcer in rats [37]. Extract of acai seed attenuated experimental colitis in rats via the TLR-4/Cox-2/NF-k*β* pathway [38]. Acai promotes jejunal tissue regeneration by enhancing the antioxidant response in 5-fluorouracil-induced mucositis [39]. Aqueous extract of acai increases blood flow acutely in rats [40]. Acai reduces the inflammatory response induced by antipsychotic drug olanzapine in macrophage cells [41]. Acai supplementation reduced hepatic oxidative stress of dams fed high-fat diet and increases antioxidant enzymes' gene expression in the offspring [42].

Inflammatory or ulcerating intestinal diseases can result in leakiness of the gut barrier [43], whereas citrus flavonoids can protect the intestinal barrier and protect against nonsteroidal anti-inflammatory drug-induced small intestine injury by promoting autophagy in vivo and in vitro [44–46]. Total citrus flavonoids attenuate nonalcoholic steatohepatitis via regulating the gut microbiota and bile acid metabolism in mice [47]. Citrus flavonoid eriocitrinon dose-dependently attenuated chemically induced tonic visceral nociception and acute phasic thermal nociception in incisional nociceptive hyperalgesia mice via the opioid receptor and GABAA receptor-mediated mechanism [48]. Consumption of citrus flavonoids for 12 weeks reduced the low-density lipoprotein cholesterol (LDL) level and waist circumference in healthy subjects [49] and reduced oxidation of LDL in naive cardiovascular subjects [50].

Hypomagnesemia is common in patients with gastrointestinal losses and other diseases [51] that can alter gut microbiota and leads to depressive-like behavior [52]. Administration of magnesium sulfate effectively attenuated the pneumoperitoneum-related hemodynamic instability during gastrointestinal laparoscopy and improved postoperative pain at serum magnesium concentrations above 2 mmol/L [53]. Magnesium citrate plus sodium picosulfate administration is effective and safe for colon cleansing [54]. Magnesium sulfate attenuates hypoxia-induced lethality and oxidative damage in mice [55]. Altered intestinal motility, secretion, absorption, and gastrointestinal transit time (GITT) are characters of gastrointestinal complications, whereas magnesium can control gastrointestinal smooth muscle contraction through the *α*-adrenoceptor antagonist pathway [56].

Vitamin C (ascorbic acid, ascorbate) deficiency is a character of patients with gastric disease, peptic ulcer, and ulcer hemorrhage due to decreased absorption, insufficient intake, increased metabolic requirement, and rapid destruction of vitamin C in the GI tract [57–59] that can be reversed by *H. pylori* eradication and worsened by proton pump inhibitor therapy. Dietary supplements of vitamin C protect gastric corpus from oxidative damage to the gastric mucosa by scavenging free radicals and attenuating the *H. pylori-*induced inflammatory cascade and decrease incidence of bleeding from peptic ulcer disease [60]. Vitamin C alleviates acute enterocolitis in *Campylobacter jejuni*-infected mice with the anti-inflammatory effects extended to extraintestinal compartments including the liver, kidneys, and lungs [61].

A meta-analysis of randomized controlled trials (RCTs) suggests that peppermint oil is safe and effective in improving global IBS symptoms and abdominal pain [15]. A network meta-analysis also ranked peppermint oil as the most effective supplement for IBS compared to soluble fiber, antispasmodic drugs, and gut-brain modulators [14]. However, other RCTs showed no significant effect of peppermint oil on IBS symptoms [20]. Similarly, while one study showed *Aloe vera* extract was effective treatment of IBS [19], other RTCs showed no effect of *Aloe vera* on IBS [16–18, 62, 63]. The cause of the discrepant efficacies between different studies remains unknown but may be due to variations in quality, quantity, and absorption route of bioactive ingredients or due to variation in CGS subtype and severity, age, gender, comorbidity, and premedical treatment. Understanding the role of these potential influencing factors could help design better intervention strategies.

The aim of this randomized placebo-controlled double-blind trial was to evaluate the safety and efficacy of ColonVita on the chronic gastrointestinal symptoms and the quality of life in old adults with chronic postprandial abdominal pain, indigestion, abdominal distension, anorexia, heartburn, vomiting, constipation, and chronic diarrhea, by using the gastrointestinal quality of life index (GIQLI) which is a specific measure for the evaluation of health status and treatment effectiveness for adults with chronic gastrointestinal condition [64]. GIQLI has been validated as a reliable scale to evaluate adults with chronic gastrointestinal problems among the Chinese-speaking population [65].

## 2. Materials and Methods

### 2.1. Participants

#### 2.1.1. Inclusion Criteria, Recruitments, and Sample Size Estimation

The recruitment of the participants occurred between November 18, 2011, and December 25, 2011, and the follow-up observation was completed on March 31, 2012, at Jiaxing Lu Community Health Service Center, Lujiazui, Shanghai, China. Participants were recruited by self-referral in response to media coverage and word of mouth. All study procedures were conducted in accordance with the Helsinki Declaration of 1975, and written informed consent form was obtained from all participants prior to enrollment into the study.

Subjects who met the first and one of the other two following criteria were eligible for the study. Inclusion criteria: (1) 50 years or older male or female; (2) stomach symptoms such as abdominal pain after a meal, dyspepsia, bloating, anorexia, heartburn, and vomiting etc., and (3) intestinal symptoms such as constipation, chronic diarrhea, and so on.

The estimated sample size was based on the hypothesis that the expected means ± standard deviation of the total GIQLI score in ColonVita groups would be 116.0 ± 10.0 after the intervention. The expected means ± standard deviation of each group would be about 110.0 ± 10.0 after the intervention. The *α* level was set as 0.05, and the power was set as 0.80. The calculated sample size is 45 per group, and the total sample size was 90. With an expected 10% dropout rate (*n* = 9), it is estimated that at least 100 patients would be required, with 50 participants per group.

### 2.2. Randomization and Blindness

Participants were randomly assigned to the intervention group (ColonVita) and the control group (placebo). The randomization was performed using a predetermined randomization code which was generated by a random number generator.

Trial participants and community doctors were both blinded from the treatment (double-blind trial). Of the 100 enrolled participants, 50 subjects were assigned to the ColonVita group and 50 subjects to the placebo group. All participants completed the 12-week study.

The participants received similar-looking capsules in color-coded bottles (white bottles for ColonVita and yellow bottles for placebo control). Neither the subjects nor the medical doctors, including the study principal investigator, knew the specific color code until the completion of the study. Both the ColonVita tablets and the control tablets were manufactured and supplied by Garda Vita Inc. (Costa Mesa, California, USA). Each participant was instructed to take three tablets of ColonVita after supper during the first 10 days of the study and afterwards take one tablet per day for the rest of the study. A new batch of supplements was dispensed every month during follow-up sessions. Each ColonVita tablet contains the following active ingredients: 331 mg blend of *Aloe* extract, acai tropical fruit extract, citrus bioflavonoids, peppermint leaves extract (200 : 1), 150 mg of magnesium hydroxide, and 30 mg of vitamin C. The placebo is composed of wheat flour powder.

### 2.3. Evaluation of Medical History and Severity of GI Symptoms

A medical questionnaire that includes birth of date, sex, medical history, family history, smoking, drinking habits, and current medicine use was collected from each participant. A Chinese version of the gastrointestinal quality of life index (GIQLI) was used to estimate the severity of the gastrointestinal symptoms and the quality of life which is specially designed for people with digestive system symptoms [64, 65]. The sensitivity of the GIQLI is estimated at 0.92, and the reliability is greater than 0.90.

This GIQLI scale is a 36-item survey with four aspects: (1) the core gastrointestinal digestion symptom (19 items, q1–q9, q27–q36, 0–76), (2) psychological/emotional well-being (5 items, q10–q14, 0–20), (3) physical well-being (7 items, q15–q21, 0–28), and (4) daily life and social activities (5 items, q22–q26, 0–20). Each survey question has five response options (0–4, from worst to best condition). The total cumulative score of the GIQLI scale is at a range of 0–144 points. The better the function/quality of life, the higher the score; the average score of normal people is 125.8 points.

Baseline values and changes in the GIQLI scores of GI-related symptoms were evaluated before and after the 12-week intervention. All participants were followed up each month in order to check compliance and adverse effects.

### 2.4. Statistics Analysis

EpiData 3.02 software was used for the data entry, and SPSS 20 software was used for statistical analysis. Group data were presented as the mean ± s.d. Differences between the ColonVita and placebo groups were compared using Student's *t*-test for quantitative variables with normal distribution and Mann–Whitney *U* test for variables with nonnormal distribution or the chi-square test for categorized variables. The alpha level of *P* > 0.05 was chosen as being statistically significant. All *P* values reported were 2-sided.

## 3. Results

### 3.1. Demographic Characteristics and Medical History of the Participants

There were no significant differences between the ColonVita and placebo groups in the proportions of gender (18M/32F vs. 19M/31F) (*χ*^2^ = 0.043, *P*=0.84), ages (63.05 ± 10.18 vs. 63.14 ± 8.59) (*t* = -0.047, *P*=0.962), people over 60 years of age (46.0% vs. 52.0%) (*χ*^2^ = 0.360, *P*=0.548), alcohol drinking (14.0% vs. 18.0%) (*χ*^2^ = 0.298, *P*=0.585), cardiovascular disease (46.0% vs. 42.0%) (*χ*^2^=0.162, *P*=0.687), use of medication (57.9% vs. 61.5%) (*χ*^2^ = 0.11, *P*=0.74), and the history of medication use for gastrointestinal symptoms (44.0% vs. 48.0%) (*χ*^2^=0.161, *P*=0.688) (Table 1).

### 3.2. Baseline of Gastrointestinal Quality of Life Index (GIQLI)

There are no baseline differences between the ColonVita and placebo groups in the total GIQLI score (101.12±16.87 vs. 101.80±16.48, *P*>0.05) and in the subdomain scores of the core GI symptoms (55.70±8.51vs. 56.30±8.58, *P*>0.05), psychological/emotional state (13.22±3.18 vs. 13.28±2.84, *P*>0.05), physiological function (17.82±5.42 vs. 17.68±5.15, *P*>0.05), and daily life and social activities (14.38±3.12 vs. 14.54±3.16, *P*>0.05) (Table 2).

After the 12-week intervention, no significant differences (*P* > 0.05) were found between the ColonVita and placebo groups in the total GIQLI score (114.78 ± 9.62 vs. 111.74 ± 13.01, *P* > 0.05), the core GI symptoms (63.22 ± 5.19 vs. 61.66 ± 7.33, *P* > 0.05), psychological emotional state (14.72 ± 1.98 vs. 14.26 ± 2.11, *P* > 0.05), physical function (22.04 ± 3.16 vs. 21.42 ± 3.71, *P* > 0.05), and daily life and social activity (14.80 ± 2.62 vs. 14.40 ± 2.91, *P* > 0.05) (Table 3). Although all scores of GIQLI subdomains were improved after the 12-week intervention, no difference was found between the ColonVita and placebo groups in before-after changes of score values (*P* > 0.05) (Table 3).

Stratified analysis showed no influence of gender, alcohol drinking, and use of GI medicine on the total GIQLI score in response to the 12-week interaction. However, stratified analysis showed a differential response within the age and CVD subgroups.

While no treatment difference was found in people under 60 years, the total score of GIQLI and the score of core GI symptoms were significantly improved in the ColonVita group than in the placebo group of people ≥60 years (118.09 ± 7.88 vs. 109.50 ± 16.71, *P* < 0.05; 64.61 ± 3.99 vs. 60.00 ± 8.65, *P* < 0.05, respectively) (Table 4).

Of CVD-free participants, supplement of ColonVita for 12 weeks resulted in significantly better improvement than placebo treatment in the total score of GIQLI (116.74 ± 9.38 vs. 110.10 ± 14.28) (*P* < 0.05) and in core GI symptoms at a trend level (63.11 ± 4.53 vs. 59.93 ± 8.03) (*P*=0.072), in an increased value of the total GIQLI score (17.22 ± 13.97 vs. 9.14 ± 15.06) (*P* < 0.05) and in an increased value of the core GI symptom score (10.89 ± 8.26 vs. 5.76 ± 8.23) (*P* < 0.05) that were similar between ColonVita and placebo groups with a CVD history (114.43 ± 11.62 vs. 114.33 ± 13.22) (*P* > 0.05) (12.30 ± 26.13 vs. 12.43 ± 22.76 f), (*P* > 0.05) and (5.48 ± 9.99 vs. 6.43 ± 8.59 for core GI symptoms) (*P* > 0.05) (Table 5), respectively.

Within GI medication-free participants, ColonVita supplement resulted in better improvement in the psychological/emotional well-being score at a trend level (1.73 ± 2.64 vs. 0.00 ± 2.60) (*P*=0.098), whereas a significant increase in the score value of the daily activity and social function than placebo was found after ColonVita supplement in participants with prior GI medicine treatment (1.73 ± 2.64 vs. 0.00 ± 2.60) (*P* < 0.05) (Table 6).

### 3.3. ColonVita and Placebo Differentially Improved Individual Core GI Symptoms

ColonVita treatment significantly (*P* > 0.05) improved the scores of 16 individual items of the core GI symptoms (q1–q6, q8, q9, q27–q33, and q35) compared to the scores of 10 individual items significantly improved by the placebo (*P* > 0.05) (q1–q5, q9, q27, q32-q33, and q35) (Table 7). The 6 items specifically improved by ColonVita are as follows: (1) time troubled by gurgling noises from the abdomen (q6), (2) time of having good appetite (q8), (3) time of not feeling well and eating slowly (q28), (4) time of feeling difficulty in swallowing (q29), (5) time of feeling urgent defecating (q30), and (6) frequency of diarrhea (q31).

### 3.4. Three Specific Core GI Symptoms Were Better Improved by ColonVita Supplement

ColonVita supplement resulted in better improvement in three of the 19 core GI symptoms scores than placebo in the two samples rank-sum test: (1) bowel sound trouble (q6) (56.55 vs. 44.45) (*P* < 0.05), (2) feeling unwell for eating slowly (q28) (56.57 vs. 44.47) (*P* < 0.05), and (3) feeling defecating urgent (q30) (56.20 vs. 44.80) (*P* < 0.05) (Table 8).

### 3.5. Five Core GI Symptoms Show Better Remission Rate after ColonVita Intervention

The remission rate (defined as the percentage of participants showing positive improvement after the intervention) of the core GI symptoms was generally greater in the ColonVita group than in the placebo group, and 5 of them reached significant levels, i.e., epigastria satiety (72% vs. 48%, *χ*^2^ = 6.0, *P*=0.014) (Q2), excessive hiccups (58% vs. 32%, *χ*^2^ = 6.83, *P*=0.009) (Q5), audible bowel sounds (50% vs. 28%, *χ*^2^ = 5.086, *P*=0.024) (Q6), discomfort due to slow eating (38% vs. 18%, *χ*^2^ = 4.96, *P*=0.026) (Q28), and urge to defecate (46% vs. 18%, *χ*^2^ = 9.0, *P*=0.003) (Q30) were significantly better in the ColonVita group than in the placebo group after the intervention (Table 9).

## 4. Discussion

In this double-blind placebo-controlled randomized trial, all participants completed the 12-week study and reported no side effects of ColonVita. No significant difference was found in the total score of GIQLI between ColonVita and placebo groups after the 12-week intervention, although ColonVita improved the total score of GIQLI to a greater extent than placebo (15% vs.10%). Furthermore, ColonVita supplement improved more individual items of the core GIQLI symptoms and to a greater extent than placebo. Specifically, ColonVita supplement significantly (*P*>0.05) improved the scores of 16 items of the 19 core GI symptoms compared to 10 items improved by placebo and improvement in 3 core GI symptoms scores, i.e., bowel sound trouble, feeling unwell for eating slowly, and feeling defecating urgency were significantly greater after ColonVita than after placebo intervention. ColonVita supplement also resulted in a significantly greater remission rate than placebo in 5 core GI symptoms, i.e., epigastria satiety, bowel sound trouble, excessive hiccups, feeling unwell for eating slowly, and feeling urgent to defecate were significantly greater. These results support beneficial effects of ColonVita supplement on core GI symptoms in old adults with CGS.

The significant placebo effect on the subjective GIQLI endpoints is in agreement with the recent review and meta-analysis of clinical trials that placebo intervention is associated with improvements in subjective clinical outcomes (response and remission) of gastrointestinal diseases, probably through the gut-brain interaction and a mechanism of enhanced healing expectation and conditioning [66–69].

In this study, the efficacy of ColonVita on the outcome of IGQLI is influenced by age. While no differences were found between ColonVita supplement and placebo in people under 60 years, the total score of GIQLI and the core GI symptoms score were significantly improved by ColonVita supplement in participants ≥ 60 years, indicating greater relieving effects of ColonVita in the older people.

As aging alone does not greatly impact the gastrointestinal tract or cause digestive dysfunction (including esophageal reflux, achalasia, dysphagia, dyspepsia, delayed gastric emptying, constipation, fecal incontinence, and fecal impaction) in old adults [70], the differential improvement between the age subgroups by ColonVita may be due to improvement in aging-related declines in the gut function and/or preservation/proliferation of nerve cells of the myenteric plexus that affects the surface area of the small intestine, digestive absorption, and mucosal defense to generate protective immunity and incidence of inflammation and oxidative stress [71, 72]. There are reports that putatively protective lactic acid bacteria, in general, and Bifidobacteria, in particular, were reduced in the elderly fecal flora, and the species diversity of the dominant fecal microflora increased with aging [5, 73–75].

It is also possible that ColonVita supplement may have reduced age-related adverse effects of a greater dosage of GI medication in the older group. It is known that the medication use and the associated adverse drug reactions increases with age, and gastrointestinal adverse effects of drug use are one of the most reported in the elderly [76]. Future study could evaluate if ColonVita supplement may improve age-related changes in the structure and function of the GI by either improving the intestinal flora or reducing the adverse drug reactions of the elderly.

In this study, cardiovascular disease (CVD) appeared to have influenced the outcome of ColonVita on GIQLI as ColonVita supplement significantly improved the total score of GIQLI and scores of core GI symptoms only in CVD-free group but not in those with CVD. While the mechanism is unknown, recent studies suggest that venous thromboembolism (VTE) is a common risk factor underlying both CGS and cardiovascular disease and is strongly related to older age and obesity [21, 22]. Cardiovascular disease and gastrointestinal disorders are top two major aging-associated health conditions that have greater decrements impact on functioning and well-being [77]. Because the contract and relaxation of the muscles lining the intestines to move food along the digestive tract can be weakened by venous thromboembolism (VTE) and by ischemic colitis that result in intermittent abdominal pain/discomfort, altered bowel patterns, and abdominal bloating/distension [6] and because the key active ingredients of ColonVita such as acai extract can improve blood flow in rats [40], reduce inflammatory response in macrophage cells [41], and reduce hepatic oxidative stress of dams fed high-fat diet and increases antioxidant enzymes' gene expression in the offspring [42], future studies should determine if the selective effectiveness of ColonVita on GIQLI in CVD-free subpopulation is unique to ColonVita or it is a general mechanism of dietary supplement rich in anti-inflammatory and antihemostasis potentials.

A potential link between CVD and gastrointestinal disorders [78–81] is in line with the lack of effectiveness of ColonVita on CGS in people with CGS-CVD comorbidity than in people without CGS-CVD comorbidity. It is known that myocardial ischemia and infarction can induce intestinal angina-related diarrhea, nausea, and vomiting [82,83]. Sharp and sporadic pains in the upper left side stomach and in the esophageal sphincter usually begins within an hour of eating a meal and lasts up to two hours due to the abnormal cardioelectrical activity. Acute intestinal ischemia-related pain may occur due to blood clots in intestinal arteries that usually originated in the heart by atrial fibrillation [84–87]. Although nausea is related to stomach pain, it may indicate heart diseases.

Recent genome-wide association studies (GWAS) show that missense mutations in the voltage-gated sodium channel gene *SCN5A* is a potential pathogenetic mechanism of CGS [88–94]. The SCN5A-encoded voltage-gated mechanosensitive Na^+^ channel Na_V_1.5 is expressed in human gastrointestinal smooth muscle cells and interstitial cells of Cajal. Na_V_1.5 contributes to smooth muscle electrical slow waves and mechanical sensitivity. In predominantly Caucasian irritable bowel syndrome (IBS) patient cohorts, 2-3% of patients have SCN5A missense mutations that alter the Na_V_1.5 function and may contribute to IBS pathophysiology [93]. Moreover, a greater percentage of individuals with *SCN5A* mutations had constipation-predominant CGS (CGS-C, 31%) than diarrhea-predominant CGS (CGS-D) (10%, *P*<0.05) [95]. Other studies showed that patients with irritable bowel syndrome and SCN5A mutation exhibited decreased NaV1.5 current and mechanosensitivity [96–99].

A potential heterogenous pathogenesis of the CGS could underlie the lower efficacy of ColonVita in people with CVD. Although inflammatory bowel disease (IBD) and irritable bowel syndrome (IBS) share similar CGS symptoms, they may have different pathogenesis. The GI tissues of IBS are not permanently damaged by inflammation as they are in IBD and should have a better chance of recovery after effective intervention. One extrapolation from our results is that CVD is more closely associated with IBD than with IBS. Or the discrepant efficacy of ColonVita between the no-CVD and CVD groups is due to potential more IBS patients in the no-CVD group than in the CVD group that may have more IBD patients.

This study has limitations. Due to limited resources, the subtypes of CGS, i.e., IBS and IBD were not diagnosed using endoscopy/colonoscopy, contrast radiography, magnetic resonance imaging (MRI), or computed tomography (CT). The number of participants is small for detecting overall effects of ColonVita on the GIQLI outcome. Blood and stool samples were not collected for genotyping or intestinal microflora analysis. Nevertheless, the discovery of aging, CVD-comorbidity, and CGS subtype as potential influencing factors would help design better trials in assessing herbal supplement in the management of CGS.

## 5. Conclusions

Despite that polyherb-based supplement, ColonVita did not show a significant effect on the total score of GIQLI; ColonVita supplement, however, improved 16 scores of the 19 core GI symptoms compared to 10 items improved by placebo in adults with CGS. In addition, the beneficial effects of ColonVita on GIQLI is more prominent in the elderly over 60 y of age and in volunteers without the history of CVDs. These beneficial effects of ColonVita in certain subpopulation indicate that the effectiveness of peppermint-based ColonVita on GIQLI is influenced by age, genetic background, comorbidity, CGS subtype, and medication that should be determined in further studies, so that different and more effective treatments can be developed for each of the specific subtype of CGS.

## Figures and Tables

**Table 1:** Characteristics of the study participants^a^ (*N* = 100).

	ColonVita (*n* = 50)	Placebo (*n* = 50)	*χ* ^2^	*P*
	*N* (%)	*N* (%)		
Sex: male (*N*)	18 (36.0)	19 (18.0)	0.043	0.836
Age (mean ± s.d.)	63.05 ± 10.18	63.14 ± 8.59	−0.047	0.962
Age ≥60 year	23 (46.0)	26 (52.0)	0.360	0.548
Alcohol drink	7 (14.0)	9 (18.0)	0.298	0.585
History of CVD	23 (46.0)	21 (42.0)	0.162	0.687
Medication	22 (44.0)	24 (48.0)	0.161	0.688

^a^Data are numbers of individuals (%) unless otherwise indicated. CVD, cardiovascular disease.

**Table 2:** Baseline GIQLI scores in ColonVita and placebo groups

	GIQLI (mean ± standard deviation)		*t*	*P*
	ColonVita group (*n* = 50)	Placebo group (*n* = 50)		
Total score	101.12 ± 16.87	101.80 ± 16.48	−0.204	0.839
Core symptom	55.70 ± 8.51	56.30 ± 8.58	−0.351	0.726
Psychological item	13.22 ± 3.18	13.28 ± 2.84	−0.100	0.921
Physical item	17.82 ± 5.42	17.68 ± 5.15	0.132	0.895
Social item	14.38 ± 3.12	14.54 ± 3.16	−0.255	0.799

*t*, *t*-test.

**Table 3:** Group differences in the GIQLI score and change in the value after 12-week intervention.

	GIQLI (mean ± s.d.)		*t*	*P*
	ColonVita (*n* = 50)	Placebo (*n* = 50)		
Total score	114.78 ± 9.62	111.74 ± 13.01	1.329	0.187
Core symptom	63.22 ± 5.19	61.66 ± 7.33	1.229	0.222
Psychological item	14.72 ± 1.98	14.26 ± 2.11	1.125	0.263
Physical item	22.04 ± 3.16	21.42 ± 3.71	0.900	0.370
Social item	14.80 ± 2.62	14.40 ± 2.91	0.722	0.472

Differences in total score	13.66 ± 18.52	9.94 ± 16.43	1.063	0.291
Differences in core symptom	7.52 ± 8.84	5.36 ± 7.48	1.319	0.190
Differences in psychological item	1.50 ± 3.46	0.98 ± 3.09	1.793	0.430
Differences in physical item	4.22 ± 6.13	3.74 ± 6.28	0.397	0.700
Differences in social item	0.42 ± 3.68	−0.14 ± 3.17	0.815	0.417

*t*, *t*-test.

**Table 4:** Effect of age on the GIQLI score after 12-week ColonVita intervention.

	GIQLI (mean ± s.d.)		*t*	*P*
	ColonVita (<60/ ≥ 60 = 27/23)	Placebo (<60/ ≥ 60 = 24/26)		
Total score <60, (27, 24)	113.63 ± 11.95	114.46 ± 9.65	−0.270	0.788
Total score ≥60, (23, 26)	118.09 ± 7.88	109.50 ± 16.71	2.343	0.025^*∗*^
Core symptom <60	62.11 ± 5.85	62.79 ± 6.43	−0.396	0.694
Core symptom ≥60	64.61 ± 3.99	60.00 ± 8.65	2.439	0.020^*∗*^
Psychological item <60	15.07 ± 2.40	14.42 ± 2.72	0.917	0.363
Psychological item ≥60	15.83 ± 2.27	14.65 ± 3.02	1.520	0.135
Physical item <60	21.44 ± 3.60	21.92 ± 2.64	−0.529	0.599
Physical item ≥60	22.83 ± 2.41	20.96 ± 4.49	1.841	0.073
Social item <60	15.00 ± 2.53	15.33 ± 2.32	−0.489	0.627
Social item ≥60	14.83 ± 2.92	13.88 ± 3.04	1.103	0.276

Differences in total score <60	10.93 ± 18.56	8.46 ± 16.26	0.502	0.618
Differences in total score ≥60	19.70 ± 21.82	12.42 ± 20.56	1.201	0.236
Differences in core symptom <60	7.44 ± 9.37	5.29 ± 7.64	0.892	0.377
Differences in core symptom ≥60	9.52 ± 9.53	6.73 ± 8.97	1.056	0.296
Differences in psychological item <60	0.48 ± 3.62	−0.21 ± 3.80	0.664	0.510
Differences in psychological item ≥60	3.26 ± 4.70	1.54 ± 4.58	1.297	0.201
Differences in physical item <60	2.59 ± 4.14	2.96 ± 5.18	−0.280	0.781
Differences in physical item ≥60	6.22 ± 7.48	4.46 ± 7.18	0.838	0.406
Differences in social item <60	0.41 ± 3.54	0.42 ± 2.84	−0.010	0.992
Differences in social item ≥60	0.70 ± 4.06	−0.31 ± 3.20	0.966	0.339

*t*, *t*-test. ^*∗*^, *P* < 0.05.

**Table 5:** Effect of CVD on the GIQLI score after 12-week ColonVita intervention.

	GIQLI (mean ± s.d.)		*t*	*P*
	ColonVita (CVD/non = 23/27)	Placebo (CVD/non = 21/29)		
Total score, CVD	114.43 ± 11.62	114.33 ± 13.22	0.027	0.979
Total score, no CVD	116.74 ± 9.38	110.10 ± 14.28	2.069	0.044^*∗*^
Core symptom, CVD	63.43 ± 5.97	63.29 ± 6.99	0.076	0.940
Core symptom, no CVD	63.11 ± 4.53	59.93 ± 8.03	1.841	0.072
Psychological, CVD	14.83 ± 2.57	14.14 ± 2.92	0.825	0.414
Psychological, no CVD	15.93 ± 2.06	14.83 ± 2.82	1.656	0.103
Physical item, CVD	21.91 ± 2.75	22.33 ± 4.08	−0.397^*∗*^	0.694
Physical item, no CVD	22.22 ± 3.51	20.76 ± 3.33	1.600	0.115
Social item, CVD	14.26 ± 3.12	14.57 ± 2.82	−0.345	0.732
Social item, no CVD	15.48 ± 2.16	14.59 ± 2.81	1.331	0.189

Differences in total score, CVD	12.30 ± 26.13	12.43 ± 22.76	−0.017	0.987
Differences in total score, no CVD	17.22 ± 13.97	9.14 ± 15.06	2.079	0.042^*∗*^
Differences in core symptom, CVD	5.48 ± 9.99	6.43 ± 8.59	−0.337	0.738
Differences in core symptom, no CVD	10.89 ± 8.26	5.76 ± 8.23	2.326	0.024^*∗*^
Differences in psychological item, CVD	2.00 ± 5.46	1.10 ± 5.92	0.527	0.601
Differences in psychological item, no CVD	1.56 ± 3.19	0.41 ± 2.59	1.476	0.146
Differences in physical item, CVD	5.00 ± 7.97	5.76 ± 8.01	−0.316	0.754
Differences in physical item, no CVD	3.63 ± 4.02	2.28 ± 4.23	1.225	0.226
Differences in social item, CVD	−0.17 ± 4.70	−0.86 ± 3.38	0.549	0.586
Differences in social item, no CVD	1.15 ± 2.66	0.69 ± 2.61	0.652	0.517

*t*, *t*-test. ^*∗*^, *P* < 0.05.

**Table 6:** Effect of GI medication on the GIQLI score after ColonVita intervention.

	GIQLI (mean ± s.d.)		*t*	*P*
	ColonVita (med/non = 22/28)	Placebo (med/non = 24/26)		
Total score, med	119.00 ± 8.93	114.50 ± 13.11	1.349	0.184
Total score, nonmed	113.07 ± 10.92	109.46 ± 14.36	1.044	0.301
Core symptom, med	64.77 ± 4.70	62.33 ± 8.11	1.260^*∗*^	0.215
Core symptom, nonmed	62.07 ± 5.32	60.42 ± 7.38	0.946	0.348
Psychological, med	16.23 ± 2.05	15.54 ± 2.72	0.960	0.343
Psychological, nonmed	14.79 ± 2.41	13.62 ± 2.70	1.683	0.098
Physical item, med	22.77 ± 3.59	22.25 ± 2.97	0.541	0.592
Physical item, nonmed	21.54 ± 2.72	20.65 ± 4.20	0.910	0.368
Social item, med	15.23 ± 2.27	14.38 ± 2.18	1.299	0.201
Social item, nonmed	14.68 ± 2.99	14.77 ± 3.28	−0.106	0.916

Differences in total score, med	23.95 ± 18.41	15.63 ± 17.69	1.564	0.125
Differences in total score, nonmed	7.89 ± 19.33	5.81 ± 18.38	0.406	0.687
Differences in core symptom, med	12.05 ± 8.90	8.29 ± 8.92	1.428	0.160
Differences in core symptom, nonmed	5.54 ± 8.92	3.96 ± 7.26	0.708	0.482
Differences in psychological item, med	3.59 ± 4.30	2.08 ± 4.10	1.218	0.230
Differences in psychological item, nonmed	0.32± 3.87	−0.58 ± 4.10	0.828	0.411
Differences in physical item, med	6.59 ± 6.80	5.25 ± 6.23	0.698	0.489
Differences in physical item, nonmed	2.43 ± 4.93	2.35 ± 6.12	0.055	0.957
Differences in social item, med	1.73 ± 2.64	0.00 ± 2.60	2.232	0.031^*∗*^
Differences in social item, nonmed	−0.39 ± 4.25	0.08 ± 3.42	−0.445	0.658

*t*, *t*-test. ^*∗*^, *P* < 0.05.

**Table 7:** Differential impact of ColonVita and placebo on the scores of core GI symptoms (Wilcoxon signed-rank test).

Item		ColonVita group		Placebo group	
		*z*	*P*	*z*	*P*
Q1	How often do you feel abdominal pain?	2.926	0.003^*∗∗*^	3.869	0.001^*∗∗*^
Q2	How often feels epigastria satiety?	4.526	0.001^*∗∗*^	3.734	0.001^*∗∗*^
Q3	How often feels abdominal distension?	3.523	0.001^*∗∗*^	3.611	0.001^*∗∗*^
Q4	How often with anal excessive exhaust?	4.162	0.001^*∗∗*^	3.289	0.001^*∗∗*^
Q5	How often with excessive hiccups?	3.778	0.001^*∗∗*^	3.035	0.002^*∗∗*^
Q6	How often troubled by bowel sound?	3.390	0.001^*∗∗*^	1.487	0.137
Q7	How often frequent bowel movements?	1.043	0.297	0.504	0.614
Q8	How often feels good appetite?	2.177	0.029^*∗*^	1.673	0.094
Q9	How often ill restricted your diet type?	2.183	0.029^*∗*^	2.044	0.041^*∗*^
Q27	How often feels sour regurgitation?	4.440	0.001^*∗∗*^	2.625	0.009^*∗∗*^
Q28	How often feels unwell for eating slowly?	2.997	0.003^*∗∗*^	0.043	0.965
Q29	How often have difficulty swallowing?	2.840	0.005^*∗∗*^	1.633	0.102
Q30	How often feels defecating urgent?	3.470	0.001^*∗∗*^	1.734	0.083
Q31	How often with diarrhea?	2.202	0.028^*∗*^	1.127	0.260
Q32	How often with constipation?	2.830	0.005^*∗∗*^	3.260	0.001^*∗∗*^
Q33	How often feels nausea?	3.621	0.001^*∗∗*^	3.251	0.001^*∗∗*^
Q34	How often blood in stool?	1.000	0.317	1.000	0.317
Q35	How often feels heartburn?	3.552	0.001^*∗∗*^	4.134	0.001^*∗∗*^
Q36	How often with fecal incontinence?	1.000	0.317	1.000	0.317

^*∗*^, *P* < 0.05. ^*∗∗*^, *P* < 0.01.

**Table 8:** A comparison of the ColonVita and placebo groups' core GI symptoms scores before and after the intervention (two samples' rank-sum test).

Item		ColonVita group	Placebo group	*z*	*P*
		Mean rank	Mean rank		
Q1	How often do you feel abdominal pain?	49.20	51.80	−0.500	0.617
Q2	How often feels epigastria satiety?	54.07	46.93	−1.311	0.190
Q3	How often feels abdominal distension?	52.44	48.56	−0.705	0.481
Q4	How often with anal excessive exhaust?	53.94	47.06	−1.276	0.202
Q5	How often with excessive hiccups?	54.93	46.07	−1.625	0.104
Q6	How often troubled by bowel sound?	56.55	44.45	−2.234	0.025∗
Q7	How often frequent bowel movements?	52.42	48.58	−0.706	0.480
Q8	How often feels good appetite?	52.35	48.65	−0.660	0.509
Q9	How often ill restricted your diet type?	51.20	49.80	−0.252	0.801
Q27	How often feels sour regurgitation?	54.81	46.19	−1.555	0.120
Q28	How often feels unwell for eating slowly?	56.57	44.43	−2.337	0.019^*∗*^
Q29	How often have difficulty swallowing?	53.49	47.51	−1.544	0.122
Q30	How often feels defecating urgent?	56.20	44.80	−2.227	0.026^*∗*^
Q31	How often with diarrhea?	53.09	47.91	−1.073	0.283
Q32	How often with constipation?	52.19	48.81	−0.655	0.513
Q33	How often feels nausea?	52.95	48.05	−0.939	0.348
Q34	How often blood in stool?	49.03	51.97	−1.342	0.180
Q35	How often feels heartburn?	47.61	53.39	−1.072	0.284
Q36	How often with fecal incontinence?	50.02	50.98	−0.438	0.661

^*∗*^, *P* < 0.05.

**Table 9:** Remission rate of the core GI symptoms after the intervention.

Item		ColonVita	Placebo	*χ* ^2^	*P*
		%	%		
Q1	How often do you feel abdominal pain?	40.0	42.0	0.041	0.839
Q2	How often feels epigastria satiety?	72.0	48.0	6.000	0.014^*∗*^
Q3	How often feels abdominal distension?	60.0	42.0	3.241	0.072
Q4	How often with anal excessive exhaust?	52.0	40.0	1.449	0.229
Q5	How often with excessive hiccups?	58.0	32.0	6.828	0.009^*∗∗*^
Q6	How often troubled by bowel sound?	50.0	28.0	5.086	0.024^*∗*^
Q7	How often frequent bowel movements?	34.0	30.0	0.184	0.668
Q8	How often feels good appetite?	50.0	42.0	0.644	0.422
Q9	How often ill restricted your diet type?	42.0	40.0	0.041	0.839
Q27	How often feels sour regurgitation?	60.0	42.0	3.241	0.072
Q28	How often feels unwell for eating slowly?	38.0	18.0	4.960	0.026^*∗*^
Q29	How often have difficulty swallowing?	22.0	10.0	2.679	0.102
Q30	How often feels defecating urgent?	46.0	18.0	9.007	0.003^*∗∗*^
Q31	How often with diarrhea?	30.0	18.0	1.974	0.160
Q32	How often with constipation?	40.0	28.0	1.604	0.205
Q33	How often feels nausea?	44.0	34.0	1.051	0.305
Q34	How often blood in stool?	2.0	2.0	0.001	1.000
Q35	How often feels heartburn?	40.0	54.0	1.967	0.161
Q36	How often with fecal incontinence?	2.0	6.0	1.042	0.307

^*∗*^, *P* < 0.05. ^*∗∗*^, *P* < 0.01.

## Data Availability

The data used to support the findings of this study are available from the first author and the corresponding author upon request.

## Abbreviations

APC: Annual percentage change
CD: Crohn's disease
CDC: US Center for Disease Control
CGS: Chronic gastrointestinal symptoms
CGS-C: Constipation-predominant CGS
CGS-D: Diarrhea-predominant CGS
ColonVita: A polyherbal supplement
Cox-2: Cyclooxygenase-2
CT: Computed tomography
CVD: Cardiovascular disease
DASH: Dietary Approaches to Stop Hypertension
GABAA: *γ*-Aminobutyric acid type A receptors
GITT: Gastrointestinal transit time
GIQLI: Gastrointestinal quality of life index
GWAS: Genome-wide association study
*H. pylori*: *Helicobacter pylori* (c)
IBD: Inflammatory bowel disease
IBS: Irritable bowel syndrome
LDL: Low-density lipoprotein
NF-kB: Nuclear factor kappa-light-chain-enhancer of activated B cells
NIH: National Institutes of Health
MRI: Magnetic resonance imaging
NaV1.5: Voltage-gated sodium channel V (Nav)
RCTs: Randomized controlled trials
SCN5A: Gene coding for voltage-gated sodium channel subtype 5A (NaV1.5)
TLR-4: Toll-like receptor 4
UC: Ulcerative colitis
VTE: Venous thromboembolism.
